# Childhood Physical and Sexual Abuse in Caribbean Young Adults and Its Association with Depression, Post-Traumatic Stress, and Skin Bleaching

**DOI:** 10.4172/2167-1044.1000214

**Published:** 2015-12-31

**Authors:** Caryl James, Azizi A Seixas, Abigail Harrison, Girardin Jean-Louis, Mark Butler, Ferdinand Zizi, Alafia Samuels

**Affiliations:** 1; 2; 3

**Keywords:** Skin bleaching, Childhood trauma, Depression, Young adults

## Abstract

**Background:**

The global prevalence of skin depigmentation/skin bleaching among blacks, estimated at 35%, is on the rise and is associated with a host of negative health and medical consequences. Current etiological approaches do not fully capture the emotional and psychological underpinnings of skin bleaching. The current study investigated the potential mediating role of depression, or post-traumatic stress symptoms (avoidance and hyperarousal) on the relationship between childhood physical and sexual abuse (CPSA) and skin bleaching.

**Methods:**

A total of 1226 university participants (ages 18–30 years and 63.4% female) from three Caribbean countries (Jamaica, Barbados, and Grenada) provided data for the current analysis. They all completed self-reported measures of general demographic information along with the short screening scale for posttraumatic stress disorder (DSM-IV), childhood trauma, and skin bleaching questions.

**Results:**

The prevalence of skin bleaching in our study was 25.4%. Our findings showed that individuals who bleached their skin were more likely to have been abused as children (21.6% versus 13.5%, p<0.001), were more likely to have significant symptoms of trauma (34.1% versus 24.0%, p=0.005), and were more likely to have significant depression (43.7% versus 35.1%, p=0.032). We found that trauma-related hyperarousal symptoms positively mediated the relationship between childhood physical and sexual abuse and skin bleaching (Indirect Effect=0.03, p<0.05), while avoidance (Indirect Effect=0.000, p>0.05) and depressive (Indirect Effect=0.005, p>0.05) symptoms did not.

**Conclusion:**

The presence of trauma symptoms and childhood physical and sexual abuse (CPSA) may increase the likelihood of skin bleaching. Findings suggest that further exploration is needed to ascertain if the presence of skin bleaching warrants being also screened for trauma.

## Introduction

Skin depigmentation/bleaching, the practice of using toxic cosmetic chemical agents to lighten the complexion of one’s skin, poses grave health consequences including but not limited to irreversible skin damage, skin cancer, and kidney failure [1–3]. The increasing prevalence of skin bleaching has become a global epidemic, particularly in Afro-diaspora regions such as the Caribbean [4]. Although skin bleaching is a common practice in both genders, it is most prevalent among females [4–9]. Majority of the explanations that seek to describe the root causes of skin bleaching predominantly fall within a psychosocial paradigm, where having lighter skin (a proxy for European ideals of beauty) is equated with greater attraction [4–9] or higher social status [10]. To our knowledge, none of these psychosocial explanations offer empirically based cognitive and psychological mechanisms to explain how skin bleaching is engendered and maintained and more specifically what factors might be driving persistent skin bleaching in spite of its deleterious effects to an individual’s health.

The most widely discussed psychosocial hypotheses to explain skin bleaching include: the historical effects of colonialism and enslavement and the idealization of European aesthetics, the bleaching syndrome, and self-esteem theory [3–9]. Skin bleaching was originally thought to be endemic to people of African origin as the psychological scars of colonialism and slavery made generations of blacks feel that they are inferior to whites [3]. Though the proposed historical genesis of skin bleaching for Africans is different when compared to Asians, similar feelings of white superiority and indigenous inferiority characterize the practice of skin bleaching [3]. The ‘bleaching syndrome’ couches the root of skin bleaching at the nexus of psychological, sociological and physiological factors. It states that individuals internalize being white as the ideal beauty, and if upon evaluating their skin’s complexion they believe there is disconnect between their skin and the white ideal, they become compelled to achieve the white ideal through skin depigmentation/bleaching [7]. This theory, however, does not explain the desire to engage in skin bleaching despite its harmful effects. The experience of negative physical health outcomes of skin bleaching and its continued use, implies that self preservation may be in question, leaving researchers to hypothesize, that the underlying motivation must be related to self-hate, as measured by low self-esteem [10].

The notion that self-esteem drives skin bleaching is a controversial one. Initially, it was believed that people bleach their skins because of low self-esteem [9] and dissatisfaction with their ethnicity. However, Charles found that mean self-esteem scores for both skin bleachers and non-skin bleachers were comparable, suggesting that self-esteem does not fully explain the desire to skin bleach. In fact, a later study suggests that skin bleachers may be content with their ethnic identity [11]. The self-esteem theory seems insufficient at providing a clear understanding of skin bleaching. Interestingly, studies continue to show that there are perceived rewards in skin bleaching where up to 90% report being satisfied with and continue to use skin bleaching agents despite knowledge and experience of the harmful effects [12–14]. Skin bleaching can be considered similar to other forms of harmful body modification behaviors like Body Dysmorphic Disorder (BDD) [15–21] and Eating Disorders (ED) [22]. Like BDD and ED, skin bleaching appears to have short-term reinforcing rewards, which makes discontinuing the behavior difficult [13,14]. Both BDD and ED patients share a common comorbidity---trauma, and specifically childhood trauma. Childhood trauma historically has been linked to extreme self-harm behaviors such as self-mutilation and bingeing [23,24]. Childhood trauma, along with BDD and ED are also more prevalent among females [15–24]. With studies highlighting a higher prevalence of CPSA and skin bleaching among females it is likely that there could be a gender effect. This prompted us to consider whether there may be a similar association between skin bleaching and childhood abuse.

With this proposed framework, we set out to explore whether skin bleaching had similar associations with childhood physical and/or sexual abuse. The objectives of the current study were to: 1) provide prevalence data on skin bleaching and childhood physical and sexual abuse (CPSA) within the Caribbean; 2) investigate whether psychological factors, in particular PTSD and depressive symptoms, drive the association between CPSA and skin bleaching; and 3) explore how gender may affect these associations.

## Methods

### Sample

This study is a subset of a larger survey in 26 countries worldwide examining health and behavior among undergraduates (age 18–30 years) [25]. A random sample of students was recruited from a university in Barbados (n=1400), Grenada (n=823) and Jamaica (n=800). Of the 1400 students recruited in Barbados, 582 (42%) completed the questionnaire and our study used data from the 577 students ≤ 30 years. Of this sample, 476 students from Barbados had complete data for all study variables. In Grenada, 431 of the 823 students recruited (52%) completed the survey. The sample was further restricted to the 300 students who were of West Indian origin and no older than age 30. Analyses that required anthropometric data was carried out on the subset of the 300 students in the Grenadian cohort that had measured anthropometry (n=167). Of this sample, 160 students from Grenada had complete survey data. 95% of the 800 students recruited in Jamaica participated (n= 756) and after restricting to those ≤ 30 years old, data from 701 students were analyzed. Of this sample, 591 Jamaican students had complete data for all study variables. This yielded a final sample of 1,227 students for analysis. The study was approved by the University of the West Indies ethical research board and conforms to the ethical principles of the Declaration of Helsinki.

### Measures

#### Child abuse

On a self-report measure, participants were asked whether they experienced traumatic events by indicating ‘yes’ or ‘no’ to being physically or sexually abused during childhood.

#### Skin bleaching

On a self-report measure, participants were asked whether they had ever used skin lighteners and frequency of use within the past year was assessed. Response options ranged from 1=never to 4=more than 10 times.

#### Trauma: Hyperarousal and avoidance

Short screening scale for DSM-IV posttraumatic stress disorder, a seven-item scale that assesses post-traumatic stress disorder symptoms was used. On this self-report measure, participants were asked to indicate ‘yes’ or ‘no’ on five of the symptoms assessing avoidance and numbing symptoms and the other two questions enquired of hyperarousal symptoms [26]. A score of 4 or greater on this scale was used to determine a positive case of PTSD with previous studies using this scale showing sensitivity of 80%, specificity of 97%, positive predictive value of 71%, and negative predictive value of 98% [26]. In other words, if a participant indicated yes to four or more of the questions, a PTSD diagnosis is likely [27]. In our study the Cronbach’s α coefficient of reliability was 0.76.

#### Depression

Depressive symptoms were measured using the self-report measure of Centre for Epidemiologic Studies Depression Scale – short version (CES-D – 10), which is the shortened version of the 20-item CES-D questionnaire developed by Anderson et al. (1994). It assesses depressed mood over the past week, and has been shown to be able to correctly identify clinical depression in child, adolescent and adult samples [28–30]. Each item is measured on a 4-point likert scale, ranging from rarely (less than one day=0) to most or all of the time (5 – 7 days=3). Scores on this measure range from 0 to 30, with a score of 10 or more indicating the presence of clinically significant depressive symptoms. The CES-D-10 has demonstrated sound reliability with internal consistency scores ranging from Cronbach α of 0.71 to 0.85 [28–30]. The Cronbach’s α for the current study was 0.76.

### Data analysis

The data were analyzed using IBM SPSS Statistics for Windows (Version 20.0, Armonk, NY, USA). Gender-based comparisons were made using independent samples t-tests. A series of bivariate and multivariate regression analyses were conducted to examine relationship between skin bleaching (dependent variable), gender, childhood physical and sexual abuse CPSA trauma and depression (independent variables). Mediational analyses were also conducted to examine whether trauma symptoms mediated the relationship between CPSA and skin bleaching. Finally, we conducted mediational analyses to examine whether trauma symptoms such as hyperarousal or avoidance mediates the effect of CPSA on skin bleaching.

## Results

### Descriptive statistics

Data consisted of N=1227 college aged-students (18–30 yrs.) from three Caribbean countries - Barbados, Grenada and Jamaica. Thirteen percent of the females (13.4%) and 8.7% of males reported having experienced childhood sexual or physical abuse. Approximately eighteen percent of females (17.9%) and 7.3% males reported skin bleaching. Of the total combined sample, 30.3% were in the clinically significant range of depression; with the highest prevalence in Jamaica (42%), followed by Barbados (28.9%) and then Grenada (4.8%). For skin bleaching, Jamaica had the highest prevalence (19%), then Grenada (11.9%) and then Barbados (7.6%). For child abuse, Grenada has the highest prevalence (17.6%), then Jamaica (16.9%) had and lastly Barbados (10.7%). For trauma, Jamaica reported the highest prevalence (30.2%), the second highest Grenada (27%) and Barbados (20.8%) the lowest (Table 1).

### Inferential statistics

Results indicate that child abuse (*r*=0.133, *p*<0.001), depression (*r*=0.081, *p*=0.005), and traumatic symptoms (*r*=0.095, *p*=0.001) are related to skin bleaching. The two components of trauma related symptoms were differentially related to skin bleaching-hyperarousal was significantly associated (*r*=0.11; *p*<0.001), while avoidance was not (*r*=0.019; *p*=0.501). There were gender differences in the sample with women having more depressive symptoms, trauma symptoms, more skin bleaching, and more childhood abuse (Table 2 and 3).

With regards to analyzed mediational models, trauma symptoms partially mediated the relationship between child physical and sexual abuse and skin bleaching frequency (Model 1) Table 4. In Model 2, hyperarousal symptoms partially mediated the relationship between CPSA and skin bleaching (Model 2). In Model 3, avoidance symptoms did not significantly mediate the relationship between CPSA and skin bleaching, although CPSA was associated with avoidance symptoms. In Model 4 and Table 5, depression did not significantly mediate the relationship between CPSA and skin bleaching. In Model 5, gender did not moderate the relationship between CPSA and skin bleaching.

## Discussion

The current study makes three significant contributions to the literature. First, we found a positive association between childhood (physical and sexual) abuse, depression, trauma and skin bleaching. Second, of the two components of trauma (hyperarousal and avoidance), hyperarousal partially mediated the relationship between child abuse and skin bleaching frequency. Third, we found gender differences with females showing more depressive symptoms, trauma symptoms, frequent skin bleaching and more childhood abuse. Lastly all three Caribbean countries showed evidence of skin bleaching. Some of these findings are consistent with the literature, while other findings add new theoretical and clinical understanding surrounding the role childhood physical and sexual abuse plays in the use and frequency of skin bleaching and whether trauma and/or depression mediate this relationship.

Our finding that a significant relationship exists between childhood physical and sexual abuse, depression and skin bleaching, holds clinical and public health utility for the way we treat and address the growing prevalence of skin bleaching in Afro-diaspora countries [5,7,10,31–33]. Curtailing skin bleaching has become a public health goal in these countries and previous public health approaches [17,32,33] have not been able to successfully stem the prevalence of skin bleaching. Perhaps, the reason for this is that skin bleaching is seen as a deviant behavior, and as such treatment approaches have been tailored to address behavior modification, which rely heavily on teaching about its harmful effects by focusing on stimulus-response, overt learning principles. We are proposing that skin bleaching, as currently conceptualized, ignores deep psychological roots that go beyond stimulus-response and behavior change paradigms. Instead, we propose an alternative approach, one that explores the role of underlying emotional and psychological factors, such as depression and trauma in the initiation and maintenance of skin bleaching practices. Although not specific to skin bleaching, early studies conducted in dermatology and BDD, seem to imply the need for skin alterations is related to trauma, in particular childhood trauma [28–33].

In Model 1, we found that trauma symptoms mediated the relationship between childhood physical and sexual abuse (CPSA) and skin bleaching. Such a finding has significant clinical implications because it suggests that addressing trauma symptoms may attenuate the relationship between childhood physical and sexual abuse and skin bleaching. However, this finding only offers a crude mechanism on how global trauma operates, while ignoring the differential effects hyperarousal and avoidance (two types of trauma symptoms) have on CPSA and skin bleaching. To address this, we performed two model analyses investigating whether hyperarousal and/or avoidance symptoms mediated the relationship between CPSA and skin bleaching. We found that hyper-arousal partially mediated the relationship between CPSA and skin bleaching, while avoidance was not significantly related. Other studies, though not measuring skin bleaching, have found similar results of hyper-arousal being associated with other comorbidities such as depression and physical health symptoms [34–38].

One theory that might explain the interrelationship between CPSA, hyperarousal and skin bleaching highlights the role of emotional numbing. Previous studies indicate that emotional numbing often co-occurs with trauma and specifically hyperarousal symptoms. Emotional numbing, a way of avoiding or suppressing any intrusive thoughts or environmental triggers associated with the trauma, is generally seen as a maladaptive way of coping with trauma and hyperarousal symptoms [36–39]. Specifically, individuals while engaging in emotional numbing invest in a considerable amount of cognitive, emotional and behavioral energy to manage or avoid their hyperarousal and re-exposure symptoms [36]. There are two ways in which the individual avoids, one requires a conscious and deliberate attempt to avoid the situation that is associated with the trauma, and the other is an unconscious effort to deal with the hyper arousal states of the trauma [36]. For our study, we argue that skin bleaching, is in itself a form of emotional numbing, used at an unconscious level to manage the trauma and hyperarousal symptoms associated with CPSA.

While avoidance did not mediate the relationship between CPSA and skin bleaching, avoidance was significantly associated with CPSA, which is consistent with previous studies. Experts believe that trauma victims use avoidance behaviors to cope with painful re-experiencing symptoms. Van der Kolk and Fisher found that individuals who experienced childhood abuse reported more maladaptive coping behaviors (such as self-mutilation and bingeing) as compared to individuals who experienced abuse as adults. These findings have implications for the long-term effects of childhood abuse as well as vulnerability to other comorbidities such as depression.

Our finding that depression was associated with CPSA is consistent with previous findings. While researchers noted severe maladaptive coping behaviors being associated with CPSA [39,40], others also indicate that trauma makes the individual more vulnerable to other comorbidities such as depression [41]. Additionally, researchers have noted that adverse childhood experiences (childhood physical and sexual abuse) were significantly related to a lifetime development of depression [41].

Lastly, our finding that females were more likely to report skin bleaching, depressive symptoms, trauma symptoms and childhood abuse corroborates previous findings, suggesting that females are a vulnerable group in the development of depression, PTSD and skin bleaching [42–45]. Studies show that skin bleaching is on the rise and is more prevalent among females [5,7,10,26,27], and those with higher academic achievement [25]. Several theories have been proposed to explain why skin bleaching is more prevalent among females, these include being more sophisticated, appear more beautiful, gain more attention or attract a mate and higher social economic standing [3,5,6,42–47]. For victims of trauma, it is plausible that one of the ways to cope with these uncomfortable feelings (shame and guilt associated with trauma) would be to avoid experiencing the emotions by altering their outward appearance. This has been previously shown with attempts to alter the body through the development of an eating disorder, and self-harm to distract from or reduce the trauma. Noteworthy is that individuals aren’t aware that these behaviors are tied to these underlying feelings related to the trauma [23,24,34]. All these may predispose victims to develop body dysmorphia, which we believe skin bleaching can be, classified as such (BDD).

Skin bleaching as a variant form of body dysmorphic disorder is guided by the notion that the phenomenology of skin bleaching and Body Dysmorphic Disorder (BDD) are quite similar [21]. According to the DSM-5 BDD is “a condition in which people perform excessive, repetitive behaviors or have repetitive mental thoughts due to perceived or actual defect in their appearance”. The similarities shared between BDD and skin bleaching are: 1) the act of skin bleaching entails excessive, repetitive behaviors, despite the health consequences [1–3]. 2) various theories have been proposed which suggests that skin bleaching is driven by the perception that the one’s skin complexion is inferior, these include the white supremacy, slavery and colonialism and to some extent the self-esteem theory [3–9]. The practice of skin bleaching given the preoccupation of the skin seems no different from other forms of BDD such as a preoccupation with and aspect of the body such as weight and size as is seen in persons diagnosed with an eating disorder. Accepting that skin bleaching and BDD are phenomenologically similar allows for consideration that they might have shared or similar etiologies.

Recent studies indicate that adult survivors of physical and sexual abuse in early-childhood or adolescence were more likely to develop BDD [21]. Individuals who experienced early childhood abuse are likely to develop body distortion beliefs and attitudes, which may evolve to body dissatisfaction, guilt, shame, and self-hatred [34]. Other findings suggest that the child abuse-BDD association might be engendered by the presence of psychological disorders such as major depression, generalized anxiety disorder, post-traumatic stress disorder, substance abuse, panic disorder, personality and eating disorders [21,34,35]. Our finding that skin bleaching is positively associated with childhood abuse, depression and trauma is consistent with studies that found an association between childhood abuse and BDD.

## Limitations and Recommendations

Although interesting findings have emerged from our study, there are some limitations that should be addressed for future studies. Firstly, our study consisted of participants from tertiary level institutions, who are not representative of the general population. Secondly, given the stigma associated with reporting abuse, individuals who experience CPSA may withhold or underreport. Additionally the instrument did not allow for the age at which the individual was abused as a child, this leaves for a broad window. Thirdly, our study did not account for the effect the magnitude of the trauma has on skin bleaching. Fourthly, our study relied on self-report measures, which may have impacted reports of highly stigmatized behaviours such as skin bleaching and trauma. We recommend that future studies should include thorough assessment of frequency and magnitude of skin bleaching. Future studies should include measures of body dysmorphia and obsessive-compulsive symptoms, as they might be related to skin bleaching. Future studies should also include participants from other low- to middle-income countries to determine transcultural determinants of skin bleaching.

## Conclusion

Childhood physical and sexual abuse and its associated psychopathologies is an area that has been widely studied. Researchers have indicated that *past traumatic experiences* result in more pervasive biological dysregulation and the individual has greater difficulty regulating their internal states. Our study proposes that skin bleaching may be a way of soothing this internal dysregulation associated with CPSA. Additionally, like previous findings, we found that CPSA can result in the occurrence of other comorbidities such as post-traumatic and depressive symptoms. “These findings are timely, as previous studies have been trying to make sense of the skin bleaching epidemic but have conceptualized skin bleaching as being psycho-social in origin and while this may be so our study shows that psychopathology may play a role”. We offer a new paradigm for exploration.

## Figures and Tables

**Model 1:**
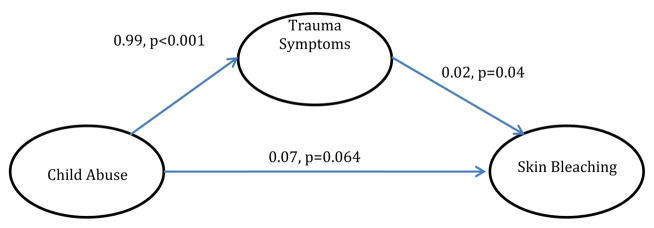
The effect of child abuse on skin bleaching, mediated by trauma (Indirect Effect=0.018, p<0.05). **Note:** There is no evidence that gender moderates the mediational relation shown above.

**Model 2:**
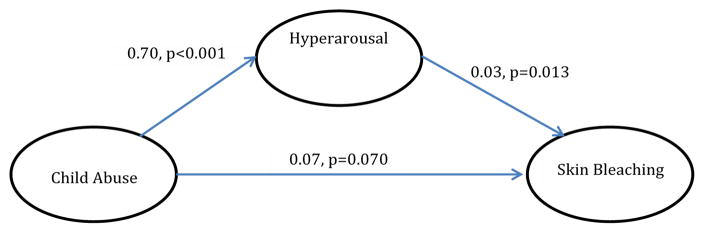
The effect of child abuse on skin bleaching, mediated by hyperarousal (indirect effect=0.020, p<0.05).

**Model 3:**
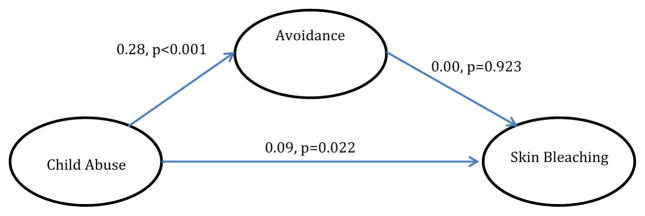
The effect of child abuse on skin bleaching, mediated by avoidance (indirect effect=0.007, p>0.05).

**Model 4:**
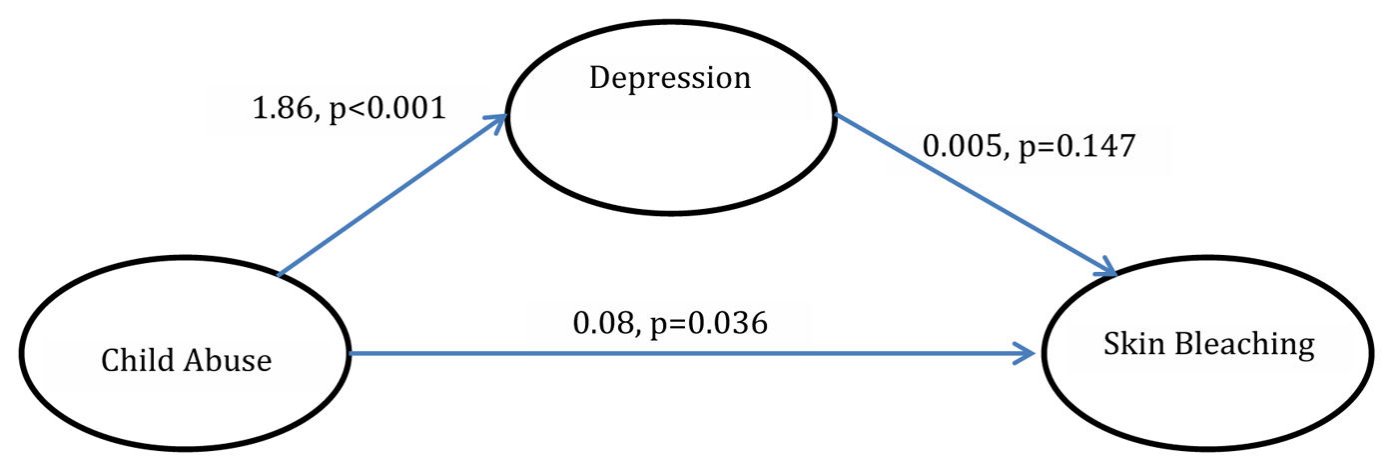
The effect of child abuse on skin bleaching, mediated by depression (indirect effect=0.009, p>0.05). There is no evidence that gender moderates the relation shown above.

**Model 5:**
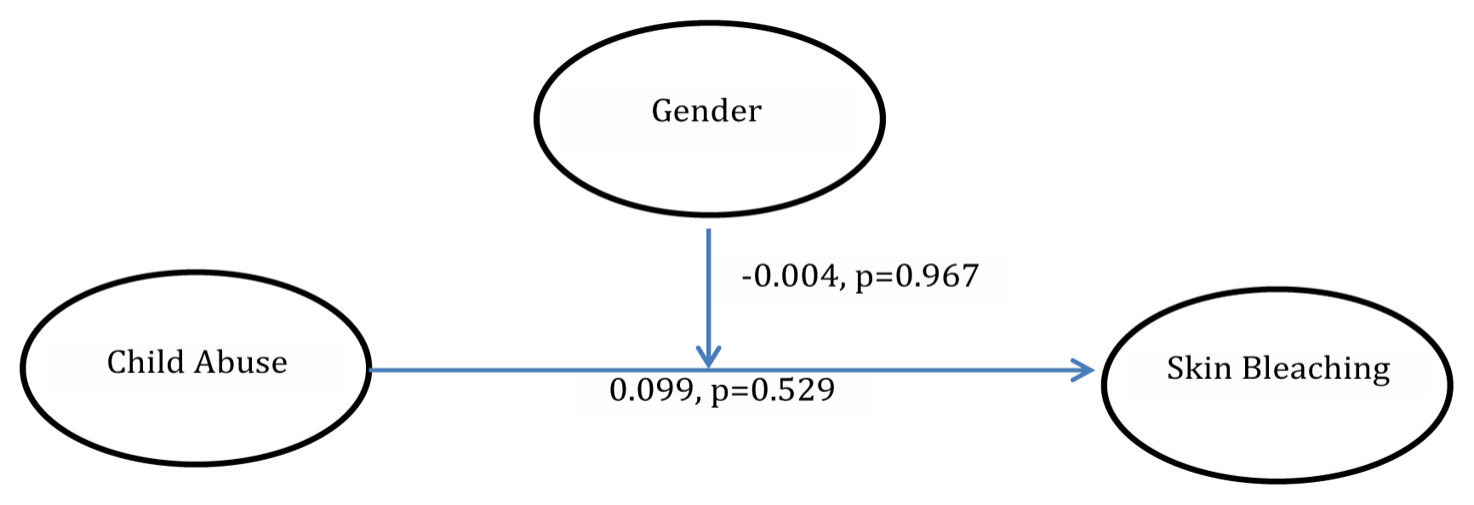
The effect of child abuse on skin bleaching, moderated by gender (interaction effect=−0.004, p=0.967).

**Table 1:** Sample descriptive for total and country of origin.

Variable	Total Sample (N=1,227)	Jamaica (N=591)	Grenada (N=160)	Barbados (N=476)	p-value
**Skin Bleaching**	0.22 (0.62)	0.31 (0.75)	0.17 (0.49)	0.11 (0.43)	<0.001
**Any Bleaching Behavior**	167 (13.6%)	112 (19.0%)	19 (11.9%)	36 (7.6%)	<0.001
**Trauma Symptoms**	2.00 (2.09)	2.32 (2.19)	2.01 (1.99)	1.61 (1.93)	<0.001
**Child Abuse (Either Physical or Sexual)**	179 (14.6%)	100 (16.9%)	28 (17.6%)	51 (10.7%)	0.017
**Depression**	8.43 (5.34)	9.22 (5.49)	8.45 (5.35)	7.46 (4.98)	<0.001
**Age**	21.02 (2.66)	20.06 (1.78)	23.09 (3.49)	21.50 (2.72)	<0.001
**Female Gender**	777 (63.4%)	451 (76.3%)	116 (73.0%)	210 (44.1%)	<0.001
**BMI**	24.10 (6.14)	23.30 (5.51)	23.67 (5.28)	25.24 (6.93)	<0.001
**Married**	25 (2.0%)	3 (0.5%)	14 (8.8%)	8 (1.7%)	<0.001

**Table 2:** Continuous variables comparisons between genders.

Variable	Female Mean (SD)	Male Mean (SD)	p-value
**Depression**	8.96 (5.60)	7.54 (4.72)	<0.001
**Trauma Symptoms**	2.11 (2.06)	1.75 (1.92)	0.002
**Hyper-arousal Subscale**	1.63 (1.62)	1.39 (1.55)	0.010
**Avoidance Subscale**	0.49 (0.69)	0.37 (0.62)	0.002
**Skin Bleaching**	0.27 (0.67)	0.12 (0.51)	<0.001
**Age**	20.89 (2.69)	21.23 (2.55)	0.028

**Table 3:** Categorical variable comparisons between genders.

Variable	Category	Female N(%)	Male N(%)	p-value
**Marital Status**	Unmarried	761 (97.9%)	440 (98.0%)	0.948
	Married	16 (2.1%)	9 (2.0%)	
**Child Abuse**	None	646 (83.1%)	401 (89.3%)	0.013
	Either Physical or Sexual Abuse	104 (13.4%)	39 (8.7%)	
	Physical and Sexual Abuse	27 (3.5%)	9 (2.0%)	
**Bleaching**	Never	638 (82.1%)	421 (93.8%)	<0.001
	1–2 Times Yearly	93 (12.0%)	12 (2.7%)	
	2–3 Times Yearly	21 (2.7%)	7 (1.6%)	
	10+ Times Yearly	25 (3.2%)	9 (2.0%)	

Note: Marital status includes only two categories

**Table 4:** Individual regressions for mediation model 1.

	B (SE)	p-value
Trauma Symptoms		
Child Abuse	0.99 (0.13)	<0.001
Skin Bleaching		
Trauma Symptoms	0.02 (0.01)	0.013
Skin Bleaching		
Child Abuse	0.09 (0.04)	0.019

**Note:** Covariates: Country of Origin, Age, BMI, and Marital Status

**Table 5:** Individual regressions for mediation model 4.

	B (SE)	p-value
Depression Symptoms		
Child Abuse	1.86 (0.34)	<0.001
Skin Bleaching		
Depression Symptoms	0.01 (0.00)	0.071
Skin Bleaching		
Child Abuse	0.09 (0.04)	0.019

**Note:** Covariates: Country of Origin, Age, BMI, and Marital Status
